# Isolated Intraventricular Metastatic Melanoma: A Case Report

**DOI:** 10.7759/cureus.83459

**Published:** 2025-05-04

**Authors:** Abhijith R Bathini, Herika Karla Negri Brito, Kliment Donev, Richard S Zimmerman

**Affiliations:** 1 Neurological Surgery, Mayo Clinic Arizona, Phoenix, USA; 2 Pathology, Mayo Clinic Arizona, Phoenix, USA

**Keywords:** external ventricular drain (evd), intraventricular melanoma, intraventricular neoplasm, melanoma, personalized oncology treatment

## Abstract

Isolated intraventricular melanoma is an infrequent disease presentation that poses unique diagnostic and management challenges. Of the few cases reported in the literature, each has a unique management plan. We present the case of a 64-year-old female with a history of gallbladder melanoma in remission after multiple cycles of chemotherapy and immunotherapy. After eight years, the patient was presented to the emergency department due to cognitive changes, memory issues, headaches, and vomiting. Imaging showed diffuse, bulky, nodular enhancement of bilateral ependyma of the lateral ventricles of unclear etiology. Systemic imaging revealed no additional sites of metastatic disease. Cytologic evaluation through multiple cerebrospinal fluid (CSF) aspirations via lumbar punctures produced unremarkable results. An external ventricular catheter was subsequently placed directly into the right lateral ventricle with no CSF able to be aspirated. Through modulated suction and aspiration, a biopsy sample was obtained through this catheter. Molecular testing of the specimen facilitated the initiation of targeted, combination chemo- and immunotherapies in conjunction with whole-brain radiation therapy. This multidisciplinary, personalized treatment plan ultimately resulted in considerable regression of her ventricular disease burden. This very rare disease presentation, along with our novel approach for tissue sampling, highlights the importance of obtaining specimens for molecular testing to guide targeted therapies that optimize patient outcomes, particularly in instances in which lumbar CSF fails to result in diagnostic yield.

## Introduction

The most common tumors of the central nervous system (CNS) are metastatic lesions, which may arise in brain parenchyma, meninges including the dura mater, or calvaria [1]. Melanoma is a known cancer with a high predisposition to metastasize to the brain [2,3]. Autopsy studies have demonstrated that 55%-75% of patients with metastatic melanoma have brain lesions, and the pattern of metastasis is typically restricted to parenchyma, leptomeninges, or dura [3-5]. Intraventricular metastatic lesions account for less than 5% of brain metastases, with isolated lesions restricted only to the ventricle being even rarer. Of these, melanoma is only a small subset, and if they appear, they are often the outcome of primary melanoma in choroid plexus or neurocutaneous melanosis [4-6].

Ependymal spread of melanoma is frequently associated with leptomeningeal disease since circulating tumor cells in the cerebrospinal fluid (CSF) can seed along the ependymal lining inside the ventricles [7]. Melanoma is also linked to BRAF V600E mutations, which are observed in 40-50% of the lesions, but a lower incidence of brain metastasis has been observed in patients who have been through BRAF-targeted therapy prior to the metastasis diagnosis [8,9].

This current paper aims to present a rare case of a patient with isolated, ependymal intraventricular melanoma who underwent a unique diagnostic workup and multi-disciplinary treatment approach.

## Case presentation

A 64-year-old female was initially diagnosed with stage IV melanoma a little over 10 years ago when she initially presented with a large gallbladder mass that was invading the liver as well as draining lymph nodes. She subsequently underwent multiple cycles of carboplatin/paclitaxel and ipilimumab, followed by hepatic artery embolization. The patient was placed on pembrolizumab for maintenance therapy with excellent response. She was in remission for approximately eight years until the onset of new symptoms. 

**Figure 1:**
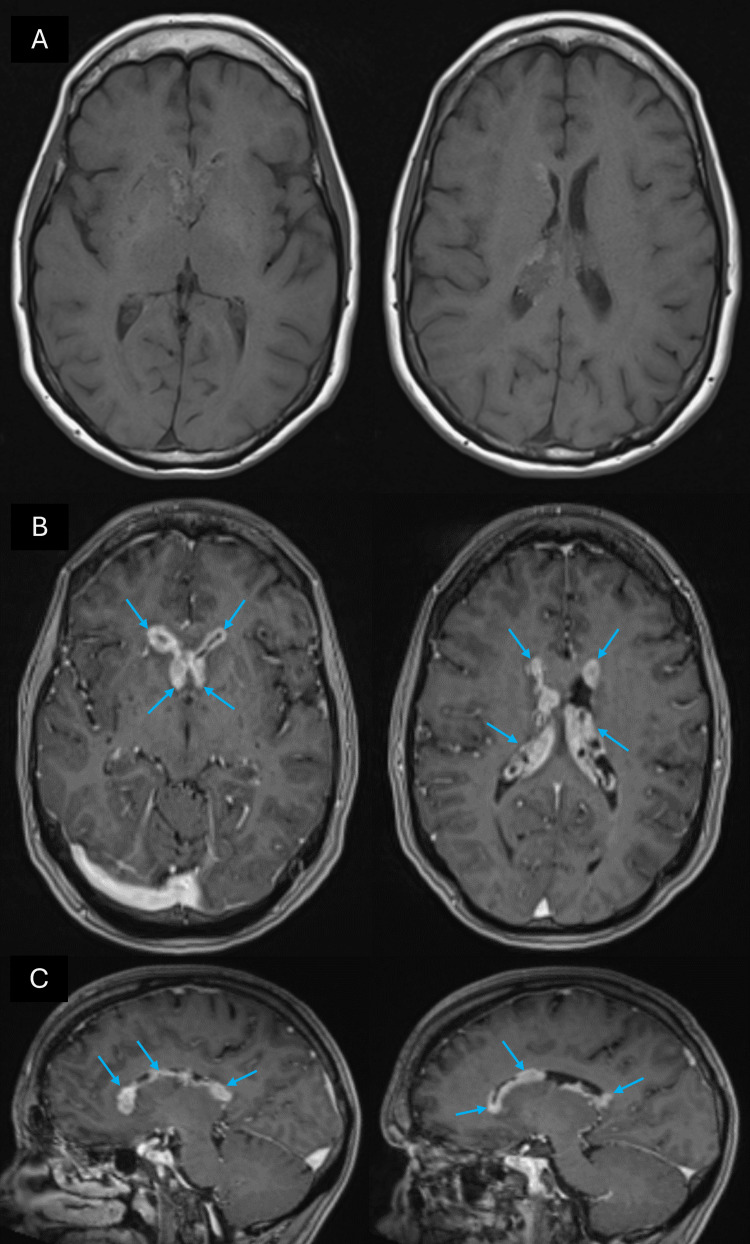
MRI at initial presentation (A) Axial T1 MRI without contrast and (B) Axial, (C) Sagittal T1 MRI with contrast showing diffuse ependymal enhancement of the lateral ventricles bilaterally (blue arrows)

The patient subsequently presented to the emergency department with a six-week history of cognitive decline, short-term memory loss, headaches, and vomiting. She underwent magnetic resonance imaging (MRI) of the brain, showing bulky enhancement of bilateral ependyma of the lateral ventricles with hemosiderin deposition (Figures 1A-1C). There was no associated ventricular enlargement or signs of periventricular edema that would have indicated hydrocephalus. The patient underwent a positron emission tomography (PET) scan revealed increased metabolic activity within the bilateral lateral ventricles corresponding to the areas of nodular ependymal enhancement that was identified on the brain MRI.

She underwent further imaging through MRI of the cervical, lumbar, and thoracic spine as well as computer tomography (CT) of the chest, abdomen, and pelvis, which were all negative for other sites of metastatic disease burden. Following imaging, the patient underwent a lumbar puncture twice with normal opening pressure. Analysis of the cerebrospinal fluid (CSF) showed lymphocytic pleocytosis and high red blood cells. Cytology was negative for any other atypical cells. Due to concern that the CSF in the lumbar cisterns was not representative of the cranial CSF sample, the decision was made to obtain CSF directly from the lateral ventricles.

A right external ventricular catheter was then placed for sampling of the lateral ventricular CSF. Very minimal CSF was able to be aspirated upon ventricular access. Therefore, through modulated suction and aspiration, we were able to obtain tissue samples through the ventricular catheter. Surgical pathology confirmed evidence of metastatic melanoma with positive cells for S100, SOX10, HMB 45, and MART-1. However, the BRAF V600E mutation was negative.

**Figure 2:**
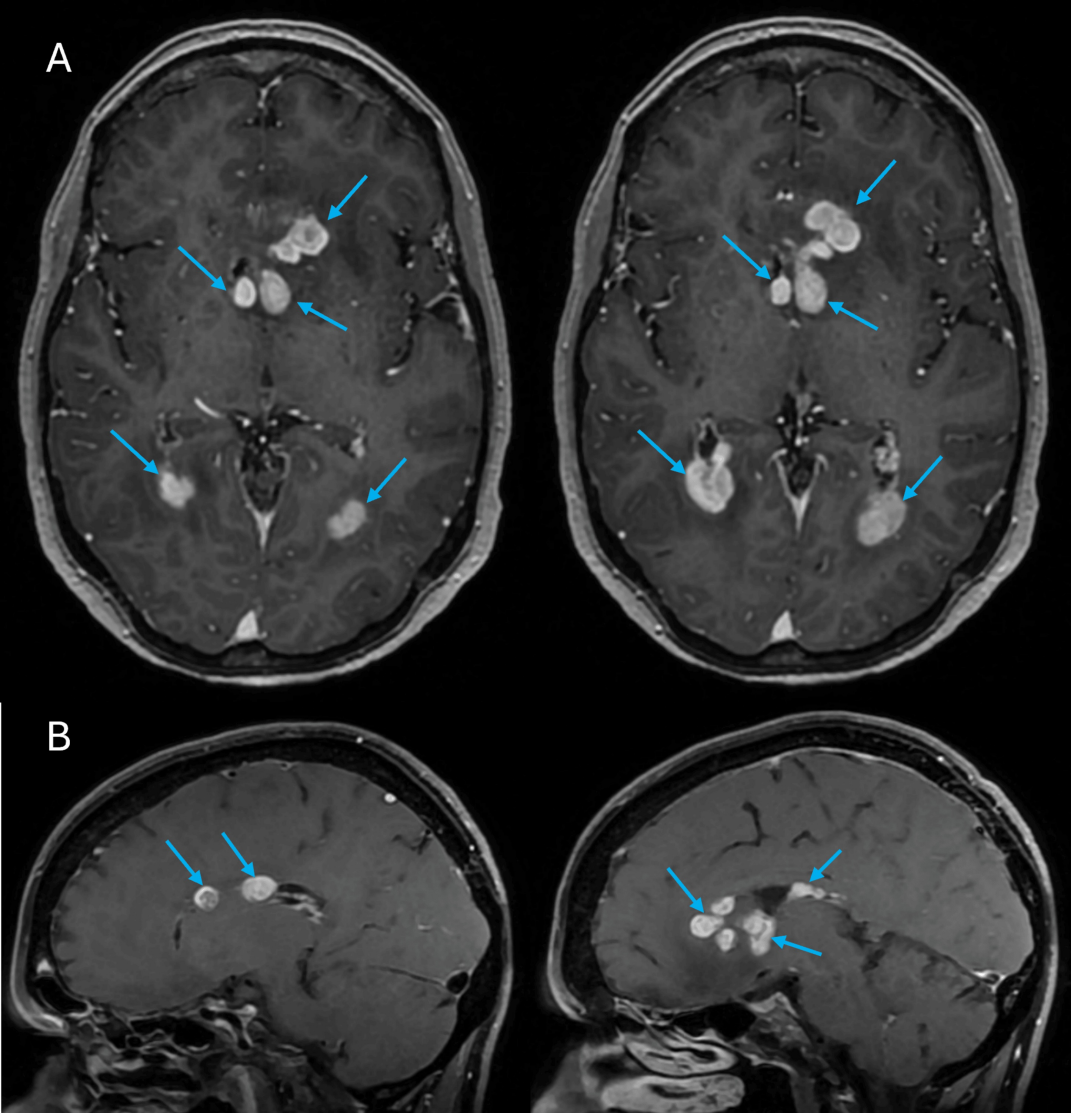
MRI scans showing interval progression of the intraventricular lesions (A) Axial and (B) Sagittal T1 MRI scans with contrast showing progression of the ependymal enhancement of the lateral ventricles bilaterally (blue arrows)

**Figure 3:**
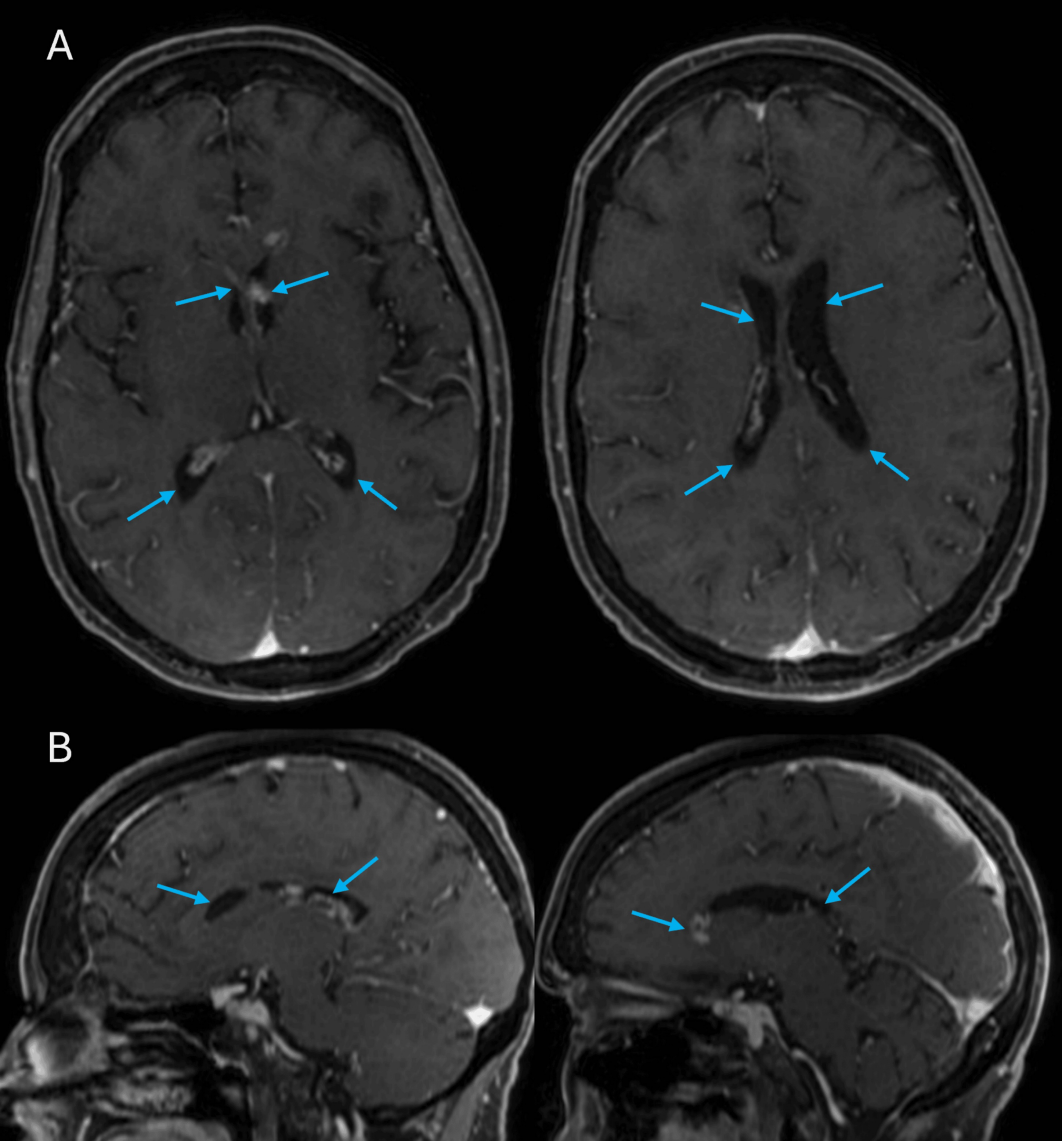
MRI scans showing considerable regression of the ventricular disease burden (A) Axial and (B) Sagittal T1 MRI scans with contrast showing substantial decrease in the size of the ependymal lesions of the lateral ventricles bilaterally (blue arrows)

Systemic staging with whole-body positron emission tomography-computed tomography (PET-CT) did not show any evidence of extracranial disease. The patient was once again started on multiple cycles of pembrolizumab with initial follow-up imaging through brain MRI showing no interval response. Therefore, ipilimumab was also added as an adjunct to the treatment plan to optimize the therapeutic response and reduce the tumor burden. She was also started on acetazolamide with symptomatic improvement in her headaches. However, four months into treatment, MRI scans of the brain showed interval progression of the intraventricular lesions (Figures 2A, 2B). The patient subsequently underwent whole brain radiation therapy (WBRT), 30 gray (Gy) in 10 fractions. This was followed by combination immunotherapy with ipilumab and nivolumab. Subsequent MRI scans showed a substantial decrease in the size of the ependymal lesions in the lateral ventricles bilaterally (Figures 3A, 3B). On the latest evaluation, seven months after the initial biopsy and two months after WBRT, the patient did start to experience some cognitive changes pertaining to memory and attention but no other neurological deficits.

## Discussion

Metastasis in the brain represents an important source of morbidity for patients, and the molecular pathway of metastatic melanomas, especially with metastasis to the CNS, is poorly understood. In the majority of CNS melanoma cases, the pattern of metastatic disease is typically leptomeningeal, intraparenchymal, or dural-based. Isolated intraventricular melanoma has rarely been reported [4,5,10].

Melanin is most prevalent within the leptomeninges in the CNS. This melanin is important to distinguish from neuromelanin which is primarily found inside neurons, such as those located in the substantia nigra pars compacta and locus coeruleus [10,11]. The melanin found in leptomeningeal disease is actually identical to that found in skin melanocytes. Within the leptomeninges, melanin is found in the highest concentrations, typically in the pia enveloping the ventrolateral medulla as well as the uppermost levels of the cervical spinal cord [4,5,10,11].

The choroid plexus has a unique developmental process as it forms from invaginated blood vessels of the pia that lie on the medial surface of the lateral ventricles [10,12]. Therefore, it is important to note that the choroid plexus is surrounded by both ependymal cells as well as pia even though it maintains a core of blood vessels. This anatomical characteristic may describe how melanocytes located initially within the pia mater can enter into the choroid plexus and, ultimately, the ependyma [10,12].

Bernstock et al. described a literature review of 14 patients with intraventricular metastatic melanoma with a median age of 42.1 years [13]. Some risk factors based on this group of patients included mucosal and/or head and neck tumors as the primary origin, male gender, neoplastic characteristics such as thick or ulcerated tumors, and acral lentiginous or nodal melanoma types [3,13]. Typically, intraventricular melanoma is still a diagnosis of exclusion, and within the ventricular system, it is most commonly encountered in the lateral ventricles. Patients often present with signs and symptoms of CNS hypertension, such as headache, nausea, vomiting, and papilledema. Obstructive hydrocephalus may also be seen due to the thick intraventricular pathological burden that hinders proper CSF flow [13,14]. Hyponatremia may be observed on routine laboratory workup. This may be caused by several variables, such as repeated vomiting, syndrome of inappropriate antidiuretic hormone secretion (SIADH), and/or adrenal or pituitary metastases [13].

**Figure 4:**
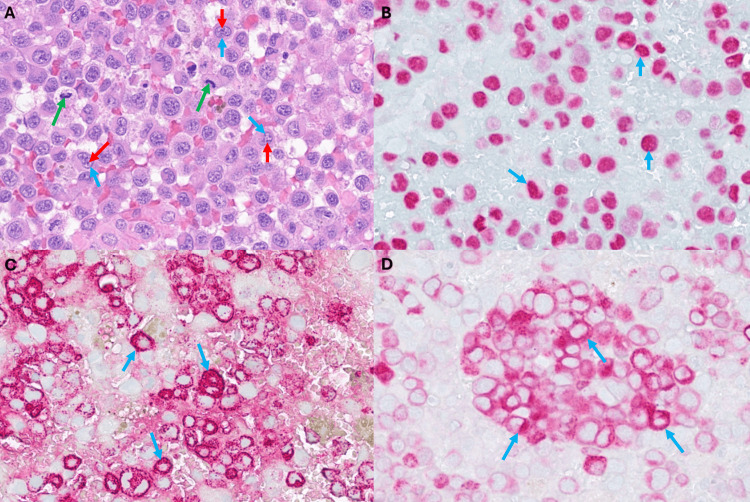
Immunohistochemical analysis of the biopsy sample The neoplasm was composed of (A) polygonal cells with irregular nuclei (blue arrows), prominent nucleoli (red arrows), and mitotic activity (green arrows). Neoplastic cells were positive for (B) SOX10 (blue arrows), (C) HMB45 (blue arrows), and (D) Mart1 (blue arrows) immunohistochemical stains.

On CT, intraventricular melanoma appears as a hyperdense contrast-enhancing mass or group of masses situated primarily in the lateral ventricles. MRI scans typically show T1 contrast-enhancing lesions lining the ventricular ependyma [10,13]. Once a tissue sample is obtained, microscopic histological analysis will show epithelioid cells with vast amounts of intracytoplasmic pigment. These cells are classically arranged in sheets and are highly positive for HMB-45 immunostaining [13]. Our biopsy results indicate neoplastic proliferation of very atypical polygonal cells with high mitotic activity associated with hemorrhage (Figure 4A). A subset of cells had plasmacytoid morphology. Further analysis showed that the neoplastic cells were positive for S100, SOX10 (Figure 4B), MART1 (Figure 4C), and HMB45 (Figure 4D) immunohistochemical stains. These findings, along with the patient's clinical history, were consistent with metastatic melanoma.

Of the cases reported in the literature, management options were quite diverse. In terms of surgical intervention, the lesions were accessed through a transcortical or interhemispheric transcallosal approach. These approaches were often performed in combination with adjuvant radiation and chemotherapy [13]. One group utilized an endoscopic exploration with biopsy through a right frontal burr hole, similar to our approach. No matter the approach for resection, outcomes were poor in cases of follow-up reporting survival [13]. If feasible, we strongly recommend utilizing a minimally invasive approach to at least obtain a biopsy of pathology so as to perform histopathological analysis that will ultimately guide the choice of specific therapies based on molecular markers. In this way, surgical time is reduced, and post-operative recovery is accelerated such that the patient may begin adjuvant therapies as soon as possible. The results of the biopsy in our patient were pivotal in navigating her adjuvant therapies and ultimate treatment response. In the future, consolidation of these rare cases would allow for analysis of the strengths and weaknesses of different management techniques as well as identification of optimal treatment strategies and patterns. 

## Conclusions

Cases of intraventricular melanoma present unique diagnostic and treatment challenges for clinicians. Our case highlights an unexpected presentation of intraventricular melanoma that was only confirmed after a biopsy that was obtained through an external ventricular drain (EVD), a first-of-its-kind management technique reported in the literature for this pathology. Of the few cases reported in the literature, surgical intervention was often associated with significant morbidity due to the deep-seated locations of these lesions as well as extensive infiltration. As a result, a combination of chemotherapy, immunotherapy, and radiation remains the mainstay of treatment of this pathology. Therefore, obtaining a biopsy sample for histopathological analysis is of utmost importance to cater to the appropriate chemo- and immunotherapies. By contributing this rare case and unique management plan to the growing body of literature, we aim to highlight the importance of implementing a multi-disciplinary team to synthesize personalized, molecular testing-based treatment plans to optimize patient outcomes.
